# The Application of Microfluidics in Traditional Chinese Medicine Research

**DOI:** 10.3390/bios15120770

**Published:** 2025-11-25

**Authors:** Shanxi Zhu, Xuanqi Ke, Yayuan Li, Zixuan Shu, Jiale Zheng, Zihan Xue, Wuzhen Qi, Bing Xu

**Affiliations:** 1 School of Chinese Materia Medica, Beijing University of Chinese Medicine, Beijing 102488, Chinaxuezihan0607@163.com (Z.X.); 2 Beijing Key Laboratory of Chinese Medicine Manufacturing Process Control and Quality Evaluation, Beijing 100029, China

**Keywords:** microfluidics, traditional Chinese medicine, quality control, organ-on-a-chip, high-throughput screening

## Abstract

Microfluidics enables precise manipulation of scarce Traditional Chinese Medicine (TCM) samples while accelerating analysis and enhancing sensitivity. Device-level structures explain these gains: staggered herringbone and serpentine mixers overcome low-Reynolds-number constraints to shorten diffusion distances and reduce incubation time; flow-focusing or T-junction droplet generators create one-droplet–one-reaction compartments that suppress cross-talk and support high-throughput screening; “Christmas-tree” gradient generators deliver quantitative dosing landscapes for mechanism-aware assays; micropillar/weir arrays and nanostructured capture surfaces raise surface-to-volume ratios and probe density, improving capture efficiency and limits of detection; porous-membrane, perfused organ-on-a-chip architectures recreate apical–basolateral transport and physiological shear, enabling metabolism-aware pharmacology and predictive toxicology; wax-patterned paper microfluidics (µPADs) use capillary networks for instrument-free metering in field settings; and lab-on-a-disc radial channels/valves exploit centrifugal pumping for parallelised workflows. Framed by key performance indicators—sensitivity (LOD/LOQ), reliability/reproducibility, time-to-result, throughput, sample volume, and sustainability/cost—this review synthesises how such structures translate into value across TCM quality/safety control, toxicology, pharmacology, screening, and delivery. Emphasis on structure–function relationships clarifies where microfluidics most effectively closes gaps between chemical fingerprints and biological potency and indicates practical routes for standardisation and deployment.

## 1. Introduction

Traditional Chinese Medicine (TCM) is a vital component of traditional medical systems and showed distinctive advantages in chronic disease management [1], cancer prevention and treatment [2], and sub-health intervention through its holistic perspective, syndrome-differentiation-based treatment principles, and preventive therapeutic philosophy [3], supported by abundant medicinal resources [4,5]. Its therapeutic efficacy primarily arises from complex multicomponent synergy and multitarget regulation. However, the chemical diversity, sophisticated compatibility, and holistic modes of action of TCM pose significant challenges in quality standardisation, bioactive component screening, and pharmacological mechanism elucidation [6,7].

Conventional analytical techniques—such as high-performance liquid chromatography, gas chromatography–mass spectrometry, and atomic absorption spectroscopy—have long underpinned TCM quality control. Yet these classical methods showed clear shortcomings with chemically complex, multicomponent systems [8], and purely chemical fingerprints often fail to predict pharmacological potency [9]. Millilitre-scale, single-parameter assays are therefore insufficient for the parallel, multidimensional evaluations that link composition with biological effects, motivating integrated microfluidic and bioassisted analytical platforms. With the rapid advancement of modern analytical technologies, microfluidic chip technology has emerged as a highly integrated, miniaturised analytical platform for TCM modernisation [10]. By precisely manipulating fluids within microfabricated channel networks at the micrometre scale, microfluidics improves key performance metrics: it reduces sample consumption by three to four orders of magnitude—from millilitre levels in traditional techniques to the nanolitre-to-microlitre range [11]—shortens analysis time from hours to minutes or even seconds [12], and achieves detection limits one to two orders of magnitude lower than conventional methods owing to high surface-to-volume ratios and short diffusion pathways [13,14]. In addition, microfluidics can build biomimetic cellular microenvironments and tightly control reaction conditions, providing a suitable platform for comprehensive analysis of complex systems. Reproducible deployment further relies on microfluidic metrology: dimensional traceability and interoperability at ports, channels, and interconnects enable leak-free, quantitative operation and cross-platform comparability [15]. In parallel, open-source, real-time analysis fosters automated droplet workflows for high-throughput screening [16], while paper-based microfluidics with tailored plasmonic nanoparticles illustrates a low-cost, field-deployable route to multiplex colourimetric detection [17].

**Figure 1:**
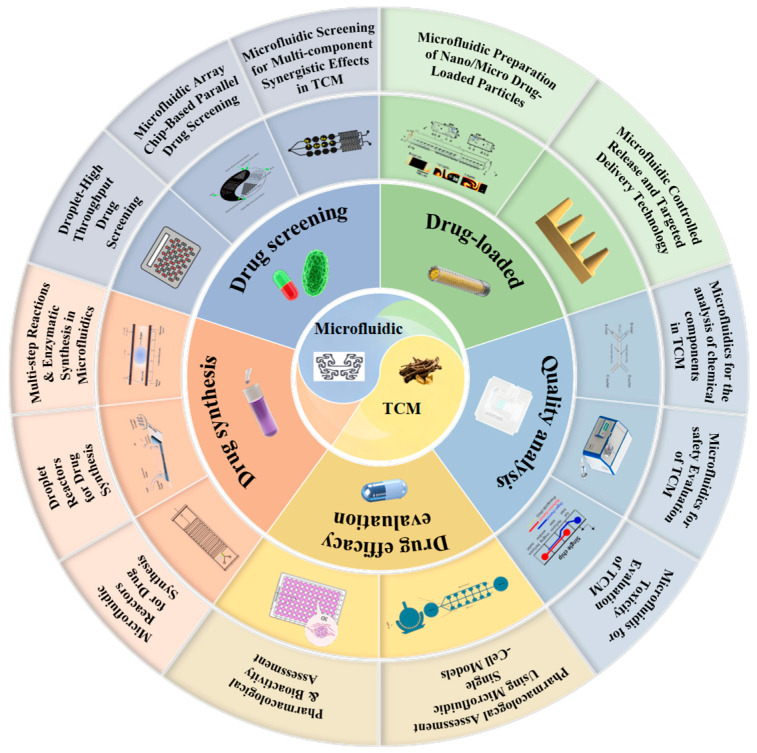
An overview of microfluidics in TCM.

In TCM research, applications of microfluidic chip technology are expanding rapidly, including efficient separation and structural identification of bioactive components [18], construction of quality control fingerprints [19,20], systematic evaluation of biological activities [21], optimisation of lead compound formulations [22], and development of novel drug delivery systems [23]. This review systematically examines the applications of microfluidic technology across the key stages of TCM research (Figure 1). Following the logical framework of TCM development, we discuss recent advances in quality analysis, safety assessment, pharmacological activity characterisation, drug synthesis, high-throughput screening, and the construction of drug delivery systems for TCM research. We aim to provide theoretical guidance and technical references for researchers in related fields, thereby supporting continued innovation and development in this interdisciplinary area.

## 2. Applications of Microfluidic Technology in TCM Research

Microfluidics enables mechanism-aware analytics for TCM under stringent sample budgets and complex matrices. To evaluate and compare platforms consistently, we anchor this section to key performance indicators (KPIs) relevant to TCM quality control: reliability/precision and reproducibility, time-to-result, throughput, sample volume, and sustainability/cost (Table 1).

### 2.1. Quality Analysis

Quality control of TCM is a critical prerequisite for ensuring clinical efficacy and medication safety. Conventional quality assessment systems primarily rely on chemical analytical methods, including quantitative determination of marker compounds and fingerprinting techniques [41], to guarantee batch-to-batch consistency and authenticity of TCM preparations. However, chemical analysis alone is insufficient to reflect the complex characteristics of TCM—which features “multi-components, multi-targets, and multi-effects”—and thus cannot fully characterise its quality attributes [42,43,44]. Consequently, establishing a more comprehensive, rapid, and accurate quality evaluation system has become an urgent requirement for TCM modernisation. In this context, the introduction of microfluidic biosensing technology offers innovative solutions for TCM safety assessment, enabling rapid detection of harmful substances including pesticide residues [26], heavy metals [27], and microbial contaminants [28], as well as real-time monitoring of potentially toxic components [45]. This advancement facilitates the construction of a more sophisticated quality control framework for TCM.

#### 2.1.1. Microfluidics for the Analysis of Chemical Components in TCM

**Figure 2:**
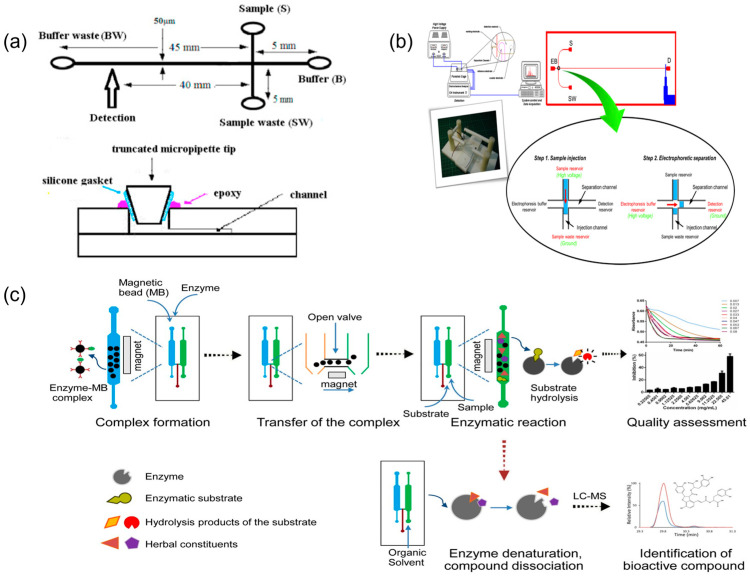
Representative microfluidic platforms for TCM chemical component analysis and bioactivity readouts. (**a**) Schematic illustration of the microfluidic chip, reprinted from [24]; (**b**) layout of the microchip electrophoresis with electrochemical detection and the diagram of electrophoresis procedures. EB, S, W, and D are the reservoirs for the electrophoresis buffer, sample solution, sample waste, and embedded Pt detector, reprinted from [46]; (**c**) schematic of microfluidic chip-based approach for quality assessment and screening of botanical drugs, reprinted from [19].

Microfluidic chip technology, with its microscale channel structures and excellent mass-transfer characteristics, can significantly enhance the efficiency of chemical-component separation and analysis, providing new technical approaches for quality control of TCM. Research has confirmed that microfluidic separation techniques, such as microchip electrophoresis, accomplish efficient separation and detection of complex TCM component systems within minutes [18]. Sun and colleagues developed a microchip capillary electrophoresis system integrated with laser-induced fluorescence detection, achieving baseline separation of two alkaloids—chelerythrine and sanguinarine—from TCM extracts within 120 s [24] (Figure 2a). This method maintained favourable linear range and detection sensitivity while substantially reducing sample and reagent consumption, demonstrating the potential of microfluidic separation as a rapid alternative to high-performance liquid chromatography in TCM analysis. In another study, researchers utilised microchip electrophoresis for rapid analysis of multiple components in *Pinellia ternata*, completing the detection process within several minutes [46] (Figure 2b). Through systematic optimisation of chip design parameters and operational conditions, this technology enables efficient extraction and analysis of multiple bioactive components in TCM, particularly trace constituents that are challenging to detect using conventional methods [27].

Furthermore, microfluidic technology showed distinct advantages in TCM sample pretreatment, enabling efficient extraction and selective enrichment of target components and thereby improving the reproducibility and reliability of fingerprint analysis. Qin et al. [47] developed a continuous laminar flow extraction microchip that achieved online solvent extraction and enrichment of active components from TCM materials. Beyond physical separation, microfluidics can also quantify medicinal component bioactivity. Li et al. [19] proposed a multiple-biomarker, dual-channel microfluidic assay to evaluate drug activity in Qi-Shen-Yi-Qi Pills on the basis of thrombin and ACE inhibition. More recently, Yang et al. [48] constructed a microfluidic SlipChip capable of generating serial nanolitre dilutions to screen pancreatic lipase inhibitors from herbal extracts within 10 min (Figure 2c). Abd Rahman et al. [25] integrated a DPPH antioxidant assay onto a centrifugal lab-on-a-disc platform to measure the radical scavenging activity of TCM-related plant extracts (e.g., *Polygonum minus*, *Syzygium polyanthum*) in under 6 min with µL-scale sample volumes. A distinctive advantage of these approaches is their capacity to integrate chemical component detection with biological activity assessment, establishing an integrated chemico-biological analysis mode. This strategy enables comprehensive evaluation of TCM quality attributes and potential therapeutic efficacy and, importantly, bridges the gap between traditional chemical testing and clinical efficacy evaluation of TCM, thereby improving the reliability of fingerprint–activity correlations in TCM analysis [19,48].

#### 2.1.2. Microfluidics for Safety Evaluation of TCM

**Figure 3:**
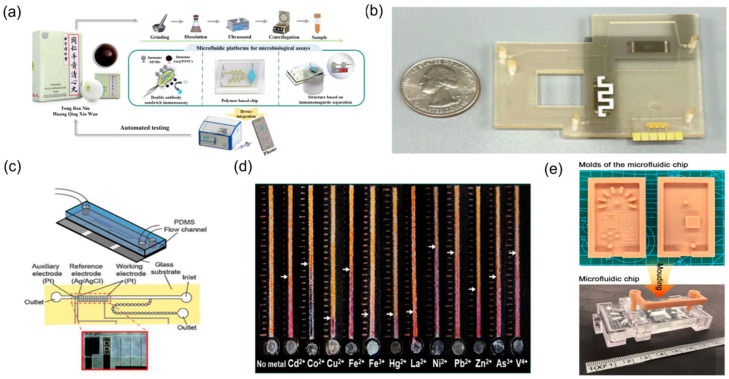
Microfluidic technologies for TCM safety testing. (**a**) Microfluidic biosensor for quantitative detection of Salmonella in TCM, reprinted from [28]; (**b**) photograph of the microfluidic system with a quarter dollar for scale, reprinted from [53]; (**c**) schematic diagram of a microfluidic device, reprinted from [52]; (**d**) microfluidic-technology-based schematic diagram for the detection of food pesticide residues, reprinted from [27]; (**e**) the developed moulds, the cured microfluidic chip, valve, and bracket, reprinted from [51].

Safety evaluation of TCM is a multidimensional, systems-level undertaking. Routine surveillance must address multiple harmful contaminants, including pesticide residues [49], heavy metal contamination [27], pathogenic microorganisms [20] and illegal additives [50] (Figure 3b). Traditional detection methods commonly entail complex procedures and prolonged turnaround times [26,51] (Figure 3c). By contrast, microfluidic biosensing provides rapid, sensitive, and field-deployable solutions for TCM safety testing. By implementing immunorecognition-, enzyme-catalysis-, or nucleic-acid-hybridisation-based biosensors on microfluidic chip platforms, highly specific detection of diverse harmful substances in TCM raw materials and preparations can be achieved.

Researchers [28] developed an integrated microfluidic optical immunosensor specifically for rapid, quantitative detection of Salmonella in TCM (Figure 3a). In this assay, a nanoenzyme catalyses on-chip colour development within the reaction chamber to amplify the signal, enabling detection of pathogenic microorganisms at concentrations as low as 90 CFU/mL within 1.5 h. Heavy metal contamination constitutes another key safety indicator. Paper-based microfluidic sensors showed particular advantages in this context. Distance-readable, paper-based microfluidic devices have been engineered by immobilising metal chromogenic reagents on paper substrates to permit visual, quantitative determination of multiple heavy metals in herbal products [27]. These devices achieve detection limits at the microgram-per-millilitre level for common analytes such as lead, mercury, and arsenic and, owing to low manufacturing cost, simple operation, and the absence of complex instrumentation, are well-suited to large-scale on-site screening of TCM products.

Furthermore, for pesticide residues and mycotoxins in TCM, enzyme inhibition principles and biorecognition elements have been integrated onto microfluidic chips to enable rapid detection of trace contaminants [52].

Comprehensive analysis indicates that microfluidic biosensor technology provides rapid, sensitive, and portable integrated solutions for TCM quality and safety testing. From pathogenic microorganisms and heavy metal contamination to illegal additives, corresponding microfluidic detection methods have been established for various harmful substances. The application of these innovative sensing technologies will powerfully support the construction of quality and safety supervision systems in TCM production and distribution, effectively ensuring the safety of TCM products through rapid on-site detection capabilities. Moreover, because most assays operate on microlitre-scale inputs and deliver minute-to-hour turnaround—often with instrument-free readout in paper-based formats—they reduce sample demand and reagent/waste burdens, thereby improving feasibility and cost-effectiveness for near-site supervision.

#### 2.1.3. Microfluidics for Toxicity Evaluation of TCM

**Figure 4:**
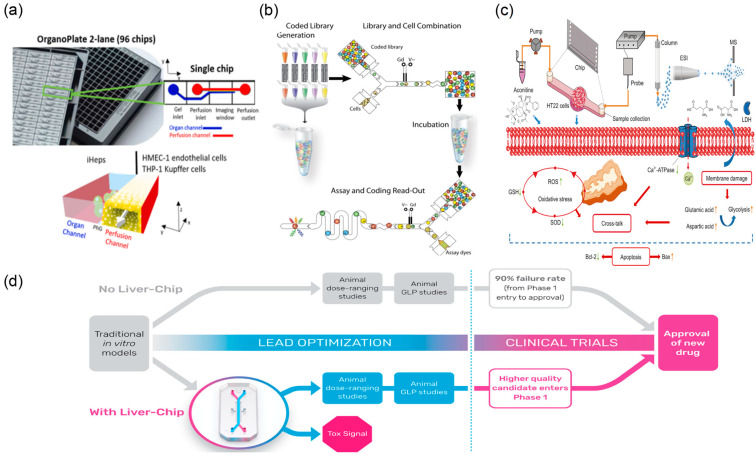
Microfluidic devices used for testing toxicity in the lab. (**a**) Image of the OrganoPlate 2-lane 96 chips and schematics of the perfusion and organ channels separated by a Phaseguide (PhG), reprinted from [30]. (**b**) Workflow of droplet-based screening. The droplet screening process consists of four steps, reprinted from [32]. (**c**) Schematic illustration of a new mechanism on neurotoxicity induced by aconitine, reprinted from [31]. (**d**) Proposed positioning of the liver chip within a typical pharma preclinical workflow, reprinted from [29].

Toxicity evaluation of TCM is a critical component of clinical medication safety. Conventional toxicity assessment primarily relies on animal experimental models; however, this approach has inherent limitations—extended study duration, high costs, and limited human relevance due to interspecies differences [54]. The emergence of microfluidic chip technology offers practical in vitro alternatives. As a representative application, organ-on-a-chip precisely reconstructs human organ microenvironments and functions in vitro, providing a more reliable platform for toxicity assessment of TCM and its active components [29,55] (Figure 4d).

In specific application studies, Bircsak et al. [30] (Figure 4a) developed a three-dimensional hepatocyte microfluidic chip integrated with a concentration gradient generator. By exposing iPSC-derived hepatocytes and supporting non-parenchymal cells to graded concentrations, they observed clear dose-dependent hepatotoxicity. Importantly, the in vitro IC_50_ values measured on-chip correlated strongly with known in vivo LD_50_ values, indicating predictive value for preclinical toxicity assessment of TCM active components. The droplet microfluidic system developed by Brouzes and colleagues enables encapsulation of individual cells within discrete microdroplets, facilitating parallel cytotoxicity screening across large-scale compound libraries [32] (Figure 4b). In this configuration, each droplet functions as an independent microreactor, allowing real-time monitoring of single-cell survival and proliferation under treatment. The platform can test thousands of conditions within a short timeframe and rapidly flag potentially toxic candidates via comparative cell viability readouts. For TCM that contains highly toxic constituents (e.g., aconitine-type alkaloids), microfluidic technology also supports mechanistic toxicology. Researchers [31] (Figure 4c) have used neuronal or cardiac tissue chip models to investigate aconitine-induced cytotoxicity, providing experimental evidence for its neurotoxic mechanisms.

Overall, microfluidic-based in vitro toxicity assessment systems can obtain TCM safety data with lower experimental costs and higher detection throughput. As organ-on-a-chip technology continues to develop and improve, microfluidic platforms will enable more comprehensive and systematic evaluation of TCM safety profiles, providing robust technical support for safety assessment and regulatory decision-making in the development of new TCM-based therapeutics. In this context, decision-relevant sensitivity requires resolving IC_50_ shifts with statistical confidence and, where available, demonstrating concordance with established in vivo LD_50_ trends. In parallel, perfused organ-on-a-chip models add human-relevant transport and metabolism, while droplet single-cell workflows increase condition throughput and reveal rare toxic phenotypes, thereby providing added predictive value beyond bulk culture.

### 2.2. Pharmacological Evaluation

The development of microfluidic technology has introduced new approaches for evaluating the pharmacological efficacy of TCM [56]. By miniaturising and integrating cellular experiments and tissue models onto chip platforms, it enables precise and efficient simulation of in vivo physiological environments [57]. In recent years, platforms have emerged, including single-cell [58] and two-dimensional cell microfluidic chips [59], three-dimensional cell-culture systems [60], organoid models [61], and organ-on-a-chip technologies [62]. Collectively, these developments have established a multi-level, multi-dimensional in vitro evaluation framework, providing support for in-depth investigation of the mechanisms of action and clinical efficacy of TCM bioactive components. Moreover, stabilised flow, nutrient delivery, and metabolite clearance across replicates improve inter-assay reproducibility and strengthen effect size estimation, thereby enhancing detection performance in pharmacological evaluations.

#### 2.2.1. Pharmacological Assessment Using Microfluidic Single-Cell Models

**Figure 5:**
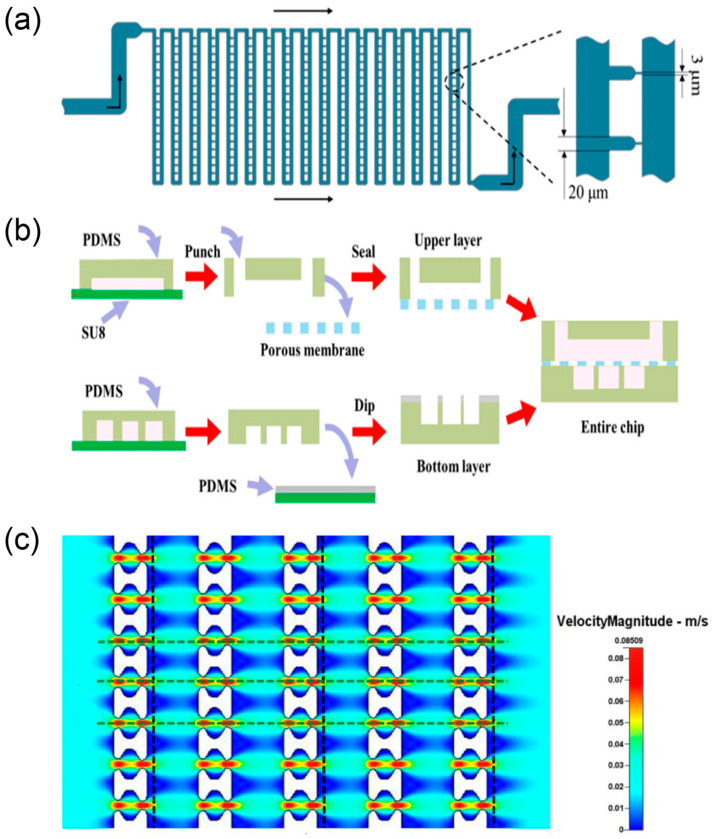
Systems for single-cell trapping and programmable droplet experiments. (**a**) Schematic diagram of the flow shortcut structure, reprinted from [63]; (**b**) the fabrication process of the multilayer microdevice, reprinted from [32]; (**c**) fluid velocity pattern formed in the 10 μm filter matrix, reprinted from [64].

Single-cell pharmacological evaluation is essential for resolving heterogeneous cellular responses to multicomponent TCM systems (Figure 5c). Conventional two-dimensional culture, which relies on population-averaged analyses, can obscure functional differences and fail to capture drug responses from rare subpopulations or defined phenotypes. Microfluidic single-cell analysis chips achieve single-cell-resolution readouts by isolating individual cells within discrete droplets or microreactors, thereby mitigating population-averaging effects on experimental results [63] (Figure 5a).

Pang et al. [64] developed microfluidic single-cell capture arrays that exploit differences in cell size and deformability to selectively isolate individual tumour cells and evaluate drug tolerance at the single-cell level. This approach is valuable for dissecting resistance heterogeneity in tumours. Because TCM preparations typically contain multiple bioactive components, microfluidic chips are well-suited to implementing complex dosing schemes and probing multicomponent synergy. Du et al. [65] constructed programmable microdroplet array platforms capable of long-term culture, time-resolved dosing, and multi-drug combinations, providing tools for systematic screening and analysis of TCM formulations. Compared with traditional single-drug assays, these microfluidic formats rapidly assess how different component combinations regulate cellular behaviours such as proliferation and survival at the microscale. Beyond conventional readouts, recent flow-glycosylation advances provide chemically defined inputs for single-cell pharmacology. Konishi et al. [66] established a continuous microfluidic glycosylation/batch-deprotection sequence that delivers panels of C-3 monodesmosidic and C-28-benzyl–C-3 saponins (including new entities) while suppressing C-3 orthoester by-product formation and minimising imidate donor usage. The availability of well-defined glycoforms with reduced side-product burden supports clean structure–activity interrogation at single-cell resolution and enables multicomponent synergy mapping by coupling library members to programmable droplet dosing and time-resolved readouts.

Applications of microfluidic chip technology in TCM pharmacological evaluation have expanded from simple proliferation assays to real-time monitoring of multidimensional pharmacological parameters—including cellular metabolism—thus providing a platform for elucidating mechanisms of action in TCM [32] (Figure 5b).

#### 2.2.2. Pharmacological and Bioactivity Assessment Using Microfluidic Organ-on-a-Chip Models

Two-dimensional culture systems lack three-dimensional architecture, complex intercellular networks, and tissue-specific microenvironments characteristic of in vivo tissues, limiting their ability to reproduce pharmacological effects of TCM in physiologically relevant settings [67]. To address this bottleneck, three-dimensional culture technologies and organoid models have rapidly evolved [68]. Co-culture systems that combine organoids with additional cell types better recapitulate tissue complexity, and microfluidic platforms provide a suitable basis for building such systems. The organoid-on-a-chip concept integrates the physiological relevance of organoids with the precise environmental control of microfluidics, connecting multiple culture units through channel networks to organise different cell types spatially and functionally. Compared with single-cell culture or organoid models alone, organ-on-a-chip technology also reproduces tissue–tissue interfaces and organ-specific physical cues (e.g., mechanical stimulation and fluid shear), and enables multi-organ cascades that emulate in vivo metabolism. These features support comprehensive pharmacological evaluation of TCM [69].

**Figure 6:**
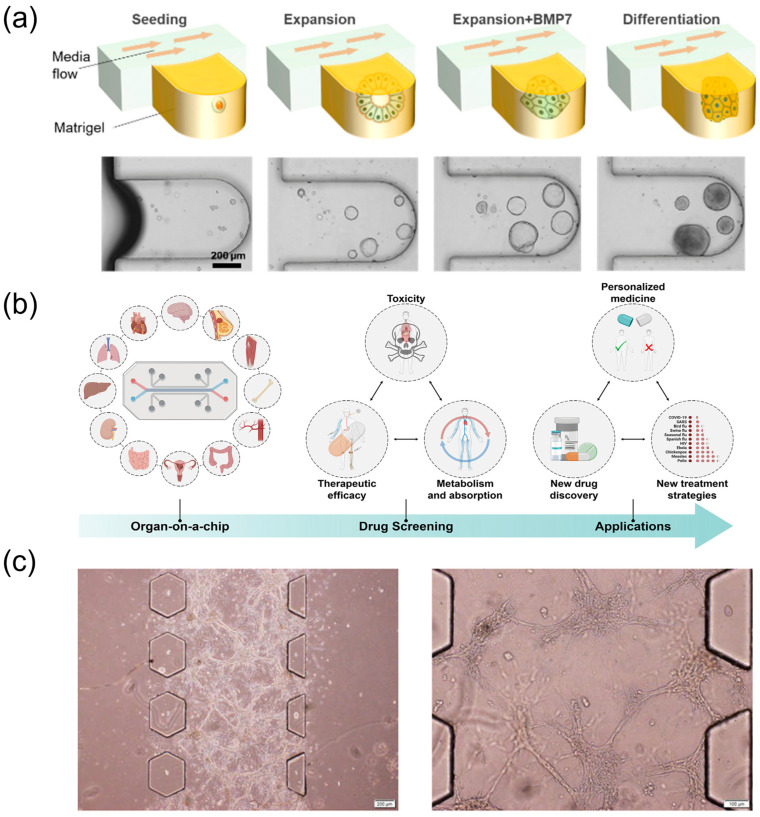
Organ-on-a-chip models for pharmacology and transport evaluation. (**a**) The mixture of mechanically dissociated cells was seeded in the MPS and cultured in the expansion medium for 4 days. Then, the cells were treated with BMP7 for an additional 2 days. The medium was changed to the differentiation medium for 5 days. MP, microphysiological system; GF, growth factors; hLO, human liver organoid; BMP7, bone morphogenetic protein 7, reprinted from [34]. (**b**) Scheme of organ-on-a-chip for drug screening, reprinted from [74]. (**c**) Growth of U251 multicellular spheroids on microfluidic chip, reprinted from [75].

In recent years, diverse microfluidic organ-on-a-chip models—liver, lung, kidney, heart and others—have been developed (Figure 6). These advances position organ-on-a-chip systems as partial replacements for specific animal study scenarios, offering human-relevant, efficient and ethically compliant evaluation methods for drug development [70]. For example, a brain-organoid-on-a-chip that coupled human brain extracellular matrix with periodic perfusion increased organoid diameter from 1.56 ± 0.44 mm to 1.84 ± 0.35 mm within 30 days and thickened cortical layers, enhancing neurogenesis [33]. In oncology, a high-throughput multi-organoid-on-a-chip enabled simultaneous perfusion testing of anticancer drugs; 48 h exposure to 10 µM toceranib reduced feline mammary tumour organoid viability and up-regulated p53/Caspase-9 while revealing necrosis in normal intestinal organoids [71]. Three-dimensional microfluidic invasion models provide physiologically relevant conditions for phenotypic validation of herbal extracts. Han et al. [72] established a tumour-invasion assay in which human glioma cells embedded in extracellular-matrix-mimicking gels were perfused with medium containing Hedyotis diffusa extract. Real-time on-chip imaging showed reduced invasion and migration, providing a microfluidics-enabled phenotypic validation of anti-glioma activity. This extract-level evidence complements organ-on-a-chip studies that incorporate hepatic metabolism and multi-organ interactions for mechanism elucidation.

For multicomponent systems such as TCM, organ-on-a-chip platforms are well-suited to probing in vivo metabolic transformation and mechanisms of action. Li and colleagues constructed a microfluidic co-culture chip with HepG2 hepatocytes in an upper chamber (liver metabolism) and tumour cells—A549 and MCF-7—in a lower chamber to compare pre- and post-metabolism effects on target cells [32]. Certain ginsenoside prototypes inhibited A549 proliferation but lost activity after liver chip metabolism, whereas the Rg3(S) prototype showed weak direct effects yet gained activity via its hepatic metabolites. Complementing this, a liver-organoid-on-a-chip microphysiological system shortened hepatocyte differentiation from ≥21 days (static) to ~11 days under flow and increased albumin and CYP3A4 expression, furnishing a rapid, metabolically competent liver module for TCM metabolism studies [34] (Figure 6a). A HUMIMIC-based multi-organoid chip integrating kidney tubuloids and liver organoids achieved 2.6-fold-higher therapeutic EV accumulation in injured kidney spheroids and a 2.1-fold increase in liver uptake, illustrating system-level efficacy and biodistribution analysis [73].

Multi-organ chips further integrate several organ models on one platform to evaluate efficacy and toxicity at a system level. A gastrointestinal–liver–kidney multi-organ chip can simulate absorption, distribution, metabolism and excretion, supporting prediction of TCM bioavailability and metabolite effects [74] (Figure 6b). Blood–brain barrier chips are useful for assessing TCM effects in the central nervous system, where permeability of active components is often uncertain; co-culture of brain microvascular endothelial cells, pericytes, and astrocytes enable quantitative evaluation of component permeability [75] (Figure 6c).

Organ-on-a-chip technology can elucidate complex mechanisms of action in TCM, with particular strengths in resolving multi-organ synergistic therapeutic effects and system-level toxic responses. In addition, these platforms advance mechanistic understanding from a modern pharmacological perspective and provide efficient screening workflows and safety assessment models for TCM drug development. As organ-on-a-chip technologies mature and standardisation progresses, their role in TCM pharmacological evaluation is likely to broaden, supporting more precise, efficient and human-relevant research.

### 2.3. Drug Synthesis

Chemical synthesis and structural modification of natural products or lead compounds are critical steps in TCM drug development [76,77]. The application of microfluidic technology in drug synthesis offers faster reactions, higher conversion efficiency, and lower material or operating costs, making it a relevant route for pharmaceutical process innovation. According to reactor configuration, microfluidic synthesis primarily comprises microchannel continuous-flow reactors and microdroplet reactors. In addition, these platforms can integrate multi-step reactions and enzymatic processes on a single chip [78].

#### 2.3.1. Microfluidic Reactors for Drug Synthesis

**Figure 7:**
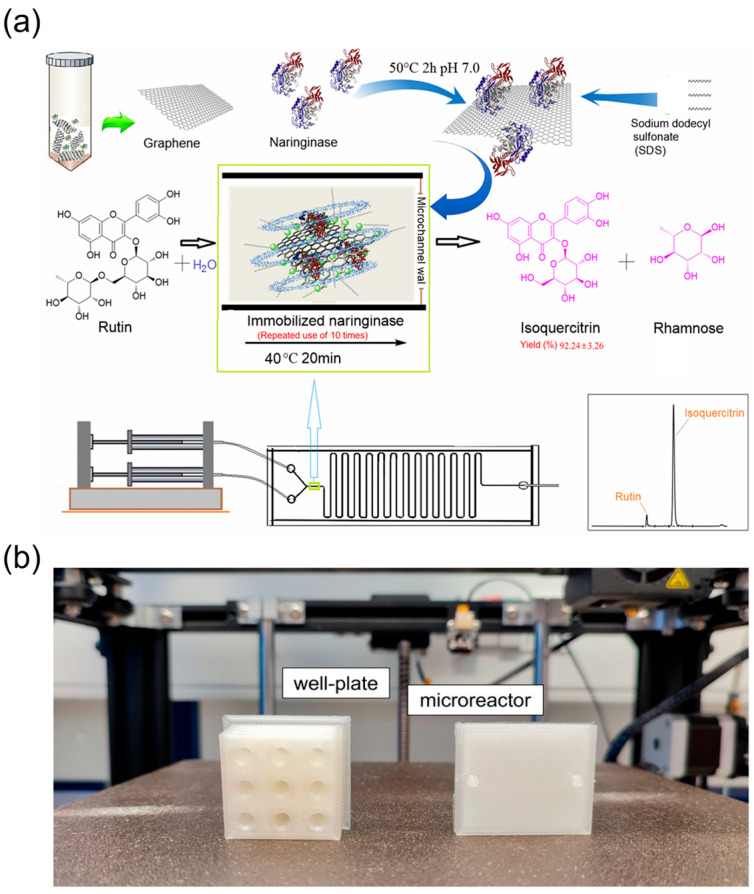
Continuous-flow microreactors for semi-synthesis of TCM monomers and derivatives. (**a**) Biosynthesis diagram of isoquercitrin in a microchannel reactor with a fluid and unsinkable immobilised enzyme, reprinted from [79]; (**b**) 3D-printed models made of natural polylactic acid (PLA). The well plate can be designed with a customised number of wells depending on the experimental needs, reprinted from [81].

In enzymatic conversion of TCM monomeric components, microchannel reactors perform efficiently. Using the flavonoid quercetin-3-O-glucoside (isoquercitrin) as an example—traditionally prepared via enzymatic hydrolysis of rutin—Gong and colleagues [79] (Figure 7a) immobilised naringinase on functionalised graphene within microchannels for flow catalysis. The system hydrolysed rutin to isoquercitrin in 20 min with 92% conversion, and the immobilised enzyme maintained stability over ten reuse cycles. Relative to batch, the reactor shortened reaction time and nearly doubled catalytic activity, illustrating the efficiency and economy of microchannel biocatalysis for TCM natural products.

For structural modification of TCM saponins, glycosylation is pivotal yet often hampered by low efficiency and by-product formation. Konishi et al. [66] transferred oleanolic acid triterpenoid saponin glycosylation to continuous-flow microreactors, enabling efficient synthesis of C-3 monoglycosides. With optimised continuous-flow conditions followed by one-step deprotection, 18 structurally diverse saponin derivatives were obtained with minimal purification. This approach delivered higher yields than batch and reduced consumption of costly glycosyl donors, improving atom economy and sustainability. Du et al. [80] recently reported a two-step continuous-flow tandem synthesis of 20 bio-amide coumarin derivatives, achieving isolated yields of 62.7–87.1% in 35 min and reducing reaction time from 24 h (batch) to 35 min (flow). Bellou et al. [81] (Figure 7b) employed a 3D-printed PLA microfluidic bioreactor in a NaDES medium for transesterification of ethyl ferulate, achieving a 23-fold increase in space–time productivity over flask reactions and retaining 53% enzyme activity after 30 days of storage.

Taken together, microfluidic reactors enable process intensification and greener synthesis of TCM monomers and natural-product derivatives, providing technical support for TCM modernisation.

#### 2.3.2. Microdroplet Reactors for Drug Synthesis

**Figure 8:**
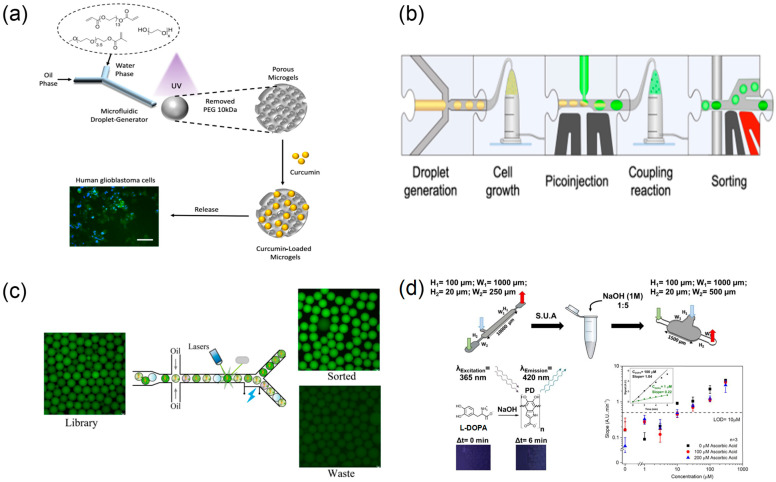
Droplet microreactors for high-throughput preparation, screening, and sorting. (**a**) Schematic illustration of microfluidics-based fabrication of porous microgels for effective delivery of curcumin to cancer cells (scale 100 µm), reprinted from [82]; (**b**) modular microfluidic workflow schematic form, reprinted from [83]; (**c**) sorting effect of microfluidic system on microdroplet, reprinted from [84]; (**d**) schematic representation of the experimental method for L-DOPA quantification, reprinted from [85].

Microdroplet microfluidic reactors achieve high-throughput, parallelised chemical synthesis by precisely encapsulating reactants within discrete microscale droplets, providing a unique technological platform for structural optimisation and formulation modification of TCM bioactive molecules [82] (Figure 8a).

Compared with microchannel continuous-flow systems, microdroplet platforms readily encapsulate individual enzyme molecules or cells in discrete droplets, enabling “one-drop-one-reaction” control. For enzymatic structural modification of TCM natural products—such as glycosidic cleavage or conjugation of compounds like forsythoside—traditional methods often require extensive condition optimisation. Microdroplet technology enables parallel screening of dozens of enzyme concentrations, reaction temperatures, and reaction times within a single experiment, thereby rapidly identifying optimal conversion conditions [83] (Figure 8b). The high surface-to-volume ratio of microdroplet reactors confers kinetic advantages. Reactions that require several hours under conventional macroscopic conditions can reach comparable conversion within minutes in microdroplet environments [85] (Figure 8d).

This throughput enables real-time screening of thousands of formulation variables. Zhu et al. [35] used colour-coded picolitre droplets to evaluate >5000 chemical combinations min^−1^ and, within a single working day, identified medium compositions that cut cell-free reaction costs four-fold. Likewise, Li et al. [84] processed ≈450,000 droplets in two weeks and isolated an Aspergillus oryzae mutant whose α-amylase activity rose 6.6-fold, exemplifying how droplet platforms rank biological activity across vast combinatorial spaces (Figure 8c). Such parallelisation accelerates optimisation of TCM bioactive small molecule synthesis. In addition, droplet platforms couple chemical synthesis directly with on-chip bioactivity assays, establishing an integrated “synthesis-to-screening” workflow at the microscale for TCM formulations.

#### 2.3.3. Integration of Multi-Step Reactions and Enzymatic Synthesis in Microfluidics

**Figure 9:**
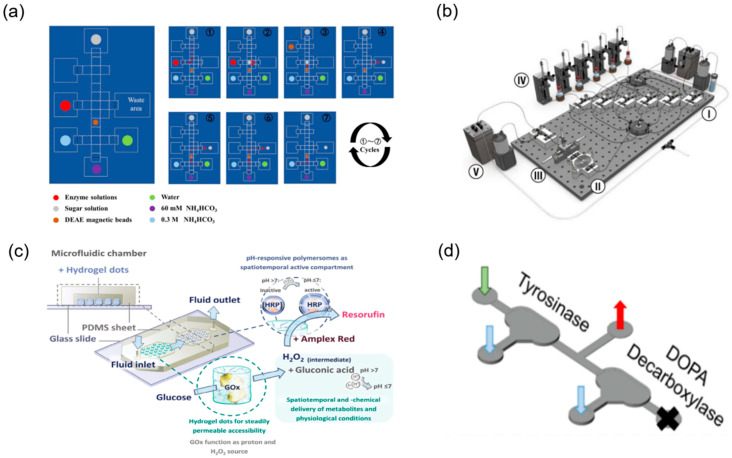
Integrated multi-step and chemo-enzymatic synthesis on microfluidic platforms. (**a**) General procedure for automatic enzymatic synthesis of tagged HMOs on the DMF device, reprinted from [78]. (**b**) Schematic of full reactor system with (I) reagent injection, (II) droplet oscillation, (III) optical sampling, (IV) phase separation, (V) waste collection, and (VI) refill modules, reprinted from [88]. (**c**) The conceptual idea of the microfluidic chip is that of a self-regulating microfluidic system, reprinted from [87]. (**d**) For the detection of dopamine, the middle outlet is sealed off and the sample is collected at the outlet, reprinted from [85].

The structural complexity of TCM necessitates the organic integration of multi-step chemical reactions with enzymatic transformations to efficiently achieve structural modification and functional optimisation of target molecules. Microfluidic technology, with its exceptional integration capabilities, enables seamless cascading of chemical reactions and biocatalytic processes on a single platform, establishing “one-pot” synthesis systems. For natural products such as triptolide that require complex multi-step modifications to obtain pharmacophore groups, the modular design concept of microfluidics provides an ideal solution [86]. By systematically connecting chemical synthesis modules (such as oxidation and condensation reactions) with biotransformation modules (such as site-specific enzymatic modifications), continuous preparation from starting materials to target products can be achieved. Research has successfully demonstrated synergistic enzyme and organic catalysis on microfluidic chips, efficiently synthesising natural product derivatives including bicyclic lactones under mild conditions [85] (Figure 9d). Wu et al. [78] combined magnetic-bead purification with modular chemo-enzymatic steps on a digital-microfluidic chip, completing an 11-step assembly of eleven human milk oligosaccharides in <3 h with isolated yields > 90% (Figure 9a). Koball et al. [87] further engineered a compartmentalised double-chamber reactor in which glucose-oxidase-generated H_2_O_2_ activated pH-responsive polymersomes loaded with horseradish peroxidase, enabling a self-regulated two-enzyme cascade that ran continuously for 12 h without loss of activity (Figure 9c). These studies exemplify how microfluidic platforms seamlessly chain chemical and biocatalytic steps, delivering high efficiency and automation for the structural diversification of complex natural products. The precise control capabilities of microfluidic technology over reaction conditions enable traditionally incompatible reaction steps to proceed sequentially within the same system [88,89,90] (Figure 9b).

### 2.4. Drug Screening

TCM systems contain abundant candidate active components and combinatorial formulations. Efficiently identifying effective components or optimising formulation strategies remains a core challenge in TCM modernisation. By miniaturising reaction systems and parallelising experimental workflows, microfluidic technology markedly increases screening throughput and provides a platform for systematic analysis of multicomponent synergy. Current efforts primarily encompass droplet-based high-throughput screening technologies, microarray-chip-based parallel screening systems, and synergistic strategies tailored to TCM formulation optimisation [91,92].

In targeted screening of TCM bioactive components, Gao [93] and colleagues developed a three-phase laminar flow microfluidic chip to evaluate selective binding of four alkaloids from Houttuynia cordata to different DNA structures. Through dynamic competitive binding experiments, this system simultaneously assessed differential affinity for tumour-associated DNA G-quadruplex targets versus normal double-stranded DNA, identifying potential anticancer components with high selectivity for tumour targets. The study achieved pharmacological evaluation and safety assessment on a single microfluidic platform, providing a paradigm for rational screening of TCM bioactive components.

The automated microfluidic screening platform developed by Schuster [94] and colleagues standardises cultivation and dosing of tumour organoids via computer-controlled microvalve arrays. The platform generates precise drug-concentration gradients and supports parallel testing of multiple drug combinations, enabling high-throughput quantitative assessment of organoid proliferation-inhibition effects.

Beyond miniaturisation and parallelisation, coupling microfluidics with artificial intelligence (AI) further improves decision quality and screening efficiency. In practice, real-time image/trajectory analysis enables automated droplet segmentation and readout extraction, which stabilises large-scale runs and reduces operator dependence [16]. AI-guided or reinforcement-learning-driven closed-loop fluidic labs can autonomously navigate multi-parameter spaces to identify optimal conditions, shortening iteration cycles and minimising reagent use [88]. At the data level, AI-driven high-throughput droplet workflows have been shown to evaluate thousands of combinations per minute and to prioritise conditions with superior performance, providing a template that can be ported to TCM contexts to predict key active monomers and synergistic pairs from microdroplet readouts (e.g., viability, enzymatic inhibition, or pathway reporters) [35]. Collectively, AI-enabled microfluidic screening offers a tractable route to rank candidates, triage follow-ups, and compress the path from exploratory assays to mechanism-aware validation.

#### 2.4.1. Droplet-Based High-Throughput Drug Screening

**Figure 10:**
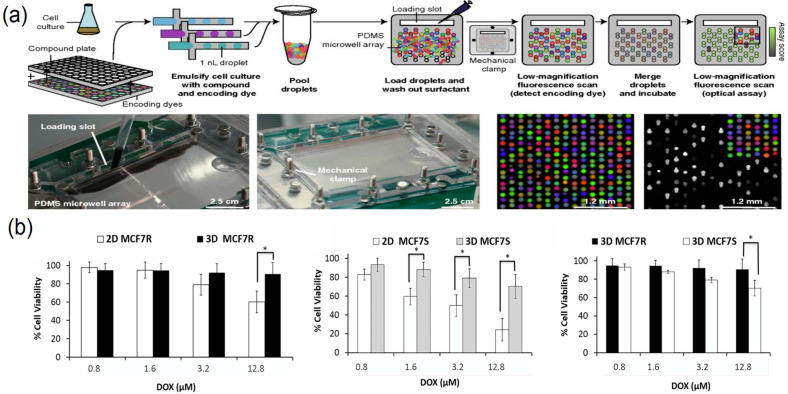
Droplet microfluidics for high-throughput drug screening. (**a**) Droplet platforms for combinatorial drug screening, reprinted from [96]; (**b**) comparing dose-dependent cytotoxicity observed with Doxorubicin in 2D versus 3D models(“*”Indicates that there is a significant difference between the results of the two groups of experiments), reprinted from [97].

Droplet microfluidics has become a core technology for high-throughput drug screening owing to low reagent consumption, rapid reaction kinetics, and high screening flux (Figure 10a,b). Its application to single-cell drug screening is particularly notable: by avoiding population averaging, it captures heterogeneous cellular responses with higher fidelity [36]. Brouzes and colleagues [95] reported an integrated droplet screening system that uses electrofusion to rapidly merge encoded droplets containing candidate drugs with single-cell droplets, enabling parallel screening of tens of thousands of drug combinations for single-cell effects within hours.

Droplet microfluidics enables high-throughput drug testing by compartmentalising reactions in pico- to nanolitre reactors and executing parallel dosing under well-defined gradients. When aligned with TCM research, droplet array formats on SlipChips have proven particularly effective for human targets. Yang et al. engineered an instrument-free serial dilution SlipChip that forms seven-point gradient droplets and co-incubates them with human pancreatic lipase. Using the TCM-derived polyphenol penta-O-galloyl-β-D-glucopyranose as a representative natural product, the platform quantified inhibitor potency with full agreement to bench assays, demonstrating a bona fide droplet-based, human-relevant screening workflow for TCM constituents [48].

Comprehensive analysis indicates that droplet microfluidic-based drug screening systems, with their outstanding characteristics of minimal reagent consumption, high detection efficiency, and large parallel throughput, demonstrate technical advantages unmatched by traditional methods in multiple application areas including single-cell pharmacological evaluation, combinatorial drug optimisation screening, and rapid drug sensitivity testing.

#### 2.4.2. Microfluidic Array Chip-Based Parallel Drug Screening

**Figure 11:**
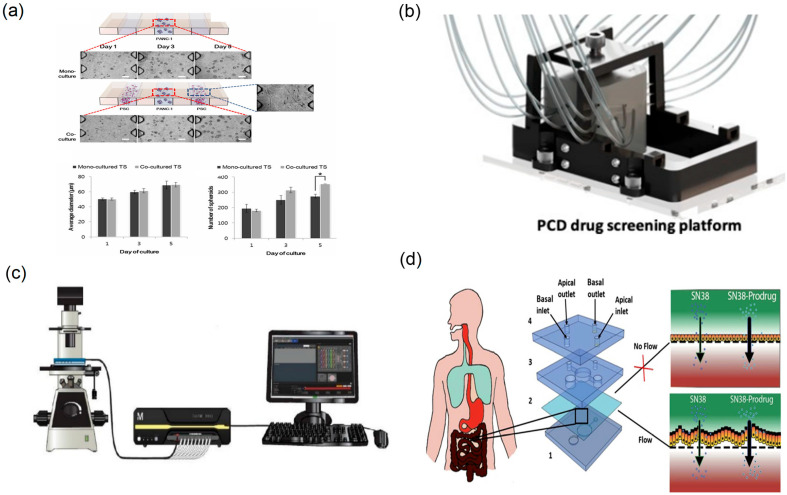
Array chip and organ chip platforms for parallel pharmacology. (**a**) Effect of PSC co-culture on growth of PANC-1 spheroids (“*”Indicates that there is a significant difference between the results), reprinted from [98]; (**b**) schematic of the fully assembled PCD drug screening platform, reprinted from [37]; (**c**) design of a CellASIC-based microfluidic platform to culture glomerular endothelial cells, reprinted from [99]; (**d**) intestine-on-a-chip microdevice design, reprinted from [48].

Beyond droplet microfluidic technology, microfluidic array chips achieve simultaneous screening of multiple experimental conditions by constructing numerous microreaction units on a single platform and they play an important role in drug evaluation research [98] (Figure 11a).

Typical microfluidic array chips employ regularly arranged microchamber or microwell arrays, enabling simultaneous execution of hundreds or even thousands of independent cell-culture and drug-treatment experiments. Compared with traditional multiwell plates, their core advantage is precise control of microenvironmental parameters for each unit, with fully automated operation realised via integrated fluid control elements (Figure 11b). The “sandwich” cell microarray chip developed by Wu and colleagues immobilised breast cancer cells within array microchambers and screened anticancer activities of natural-product extracts by fluorescence detection, achieving high-throughput, low-cost cell-level activity evaluation [37,100]. This chip adopts a “Christmas-tree” microchannel design that generates precise drug-concentration gradients in a single step while simultaneously acquiring cellular-response data at six concentrations, thereby increasing the efficiency and data density of active-component screening.

Microfluidic array chips also provide advantages for long-term culture and parallel multi-drug evaluation (Figure 11d). Beyond oncology-oriented array chips, TCM-focused microfluidic platforms using human models have enabled parallel dosing and readouts directly informative for safety and efficacy assessment. Using a plate-based, multi-channel microfluidic model lined with human renal glomerular endothelial cells (HRGECs), Qin et al. [99] (Figure 11c) exposed cells to graded concentrations of aurantio-obtusin (the major anthraquinone from Cassiae semen) in parallel, and quantified cytotoxicity (LDH release), inflammatory mediators (IL-6, TNF-α, TGF-β1, MCP-1) and tight-junction integrity (ZO-1). The data showed dose-dependent injury and barrier dysfunction, establishing a human-relevant, chip-based workflow for parallel safety profiling of TCM constituents. In a complementary human-cell study, Yang et al. [101] perfused emodin (a major anthraquinone from *Rheum palmatum*) through microchannels seeded with HRGECs under multi-dose conditions, revealing concentration-dependent nephrotoxicity and increased endothelial permeability. Beyond fluorescence imaging, researchers have also combined array chips with mass-spectrometry analysis to monitor and analyse drug metabolites online [102].

In summary, microfluidic array chips improve the efficiency and data quality of in vitro drug screening through parallelised, automated, and integrated designs [101]. In TCM active component discovery and personalised medication strategy development, array chip technology showed broad application prospects.

#### 2.4.3. Microfluidic Screening for Multicomponent Synergistic Effects in TCM

Given the numerous component types and complex targets characteristic of TCM systems, efficiently identifying active components, optimising compatibility ratios, and elucidating molecular mechanisms of synergy remain major challenges for TCM modernisation [21]. Microfluidic technology offers practical approaches to these issues. Using microfluidic chip platforms, researchers can rapidly screen combinations and concentrations of TCM extracts at the microscale while simultaneously evaluating efficacy and toxicity, achieving a “single-experiment, multidimensional data-acquisition” workflow.

**Figure 12:**
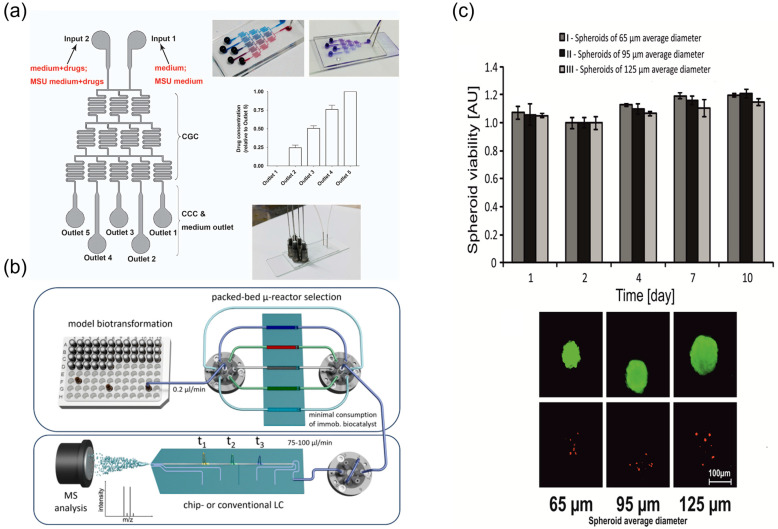
Microfluidic screening of multi-component synergy and long-term culture readouts. (**a**) Schematic graph of the integrated microfluidic 3D flowing GCC for screening, reprinted from [21]; (**b**) schematic sketch of the presented packed-bed multi-microreactor setup with automated reactor selection and online (chip)-LC/MSdetection, reprinted from [90]; (**c**) HepG2 spheroids viability during 10-day culture. Microscopic analysis (differential staining) of the viability of HepG2 spheroids, reprinted from [104].

Wang et al. [103] developed a multifunctional microfluidic chip that integrates concurrent efficacy and toxicity readouts to evaluate the “efficacy enhancement and toxicity reduction” features of TCM compatibilities. In the device, tumour cells and hepatocytes are cultured in parallel microchannels; dosing schemes for single herbs and compound formulas are precisely controlled, and on-chip, real-time monitoring of cell viability and injury markers is performed. The platform enables direct comparison of therapeutic effects and toxicities between single components and formulations, providing experimental support for TCM compatibility theory. Qian and colleagues [21] employed a microfluidic platform to evaluate the anti-gout activity of gypenoside, a monomeric component from Gynostemma pentaphyllum. A gouty-inflammation model was established on-chip via co-culture of macrophages and endothelial cells, and the regulatory effects of gypenoside on inflammatory signalling pathways were characterised (Figure 12a). Subsequent animal studies corroborated the on-chip findings, indicating that microfluidic technology can efficiently identify key bioactive molecules in TCM and elucidate their mechanisms of action.

In addition, advanced microfluidic configurations have been developed for integrated metabolic analysis and long-term cytotoxicity evaluation. A packed-bed multi-microreactor coupled with online LC-MS enables dynamic biotransformation assessment of TCM components (Figure 12b) [90], while long-term culture of HepG2 spheroids in microfluidic chips facilitates anticancer drug cytotoxicity studies, showing sustained viability over 10 days under continuous perfusion (Figure 12c) [104].

Overall, microfluidic screening technology provides a powerful technical platform for TCM active component discovery, compound formula optimisation, and synergistic mechanism research. Numerous research teams have successfully employed this technology to validate the scientific basis of classical TCM compatibility principles and discovered novel synergistic combinations, making significant contributions to advancing TCM modernisation and innovative drug development.

### 2.5. Drug Delivery and Release

Microfluidic technology has shown value in drug delivery and controlled release systems for TCM, opening avenues to enhance the therapeutic efficacy of TCM preparations [38]. Microfluidic chips provide precisely controllable platforms that support both experimental studies and manufacturable workflows for delivery of bioactive components in compound formulations. This section summarises the design and application of microfluidic drug delivery microdevices, microfluidic-based preparation of nano-/micro-drug-loaded particles, and recent advances in microfluidic controlled release and targeted delivery technologies. Accordingly, chip-based fabrication yields tighter particle-size distributions and programmable dosing, improving batch-to-batch consistency and the predictability of release kinetics, and thereby stabilising pharmacodynamic readouts.

#### 2.5.1. Microfluidic Preparation of Nano-/Micro-Drug-Loaded Particles

**Figure 13:**
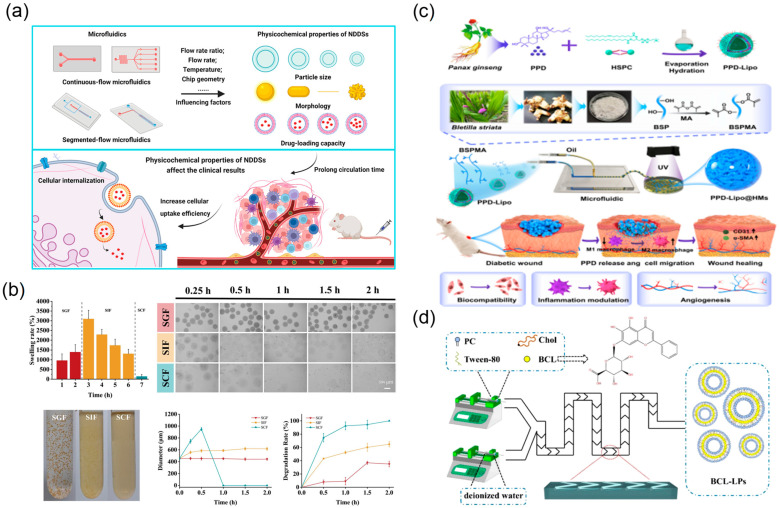
Microfluidic preparation and characterisation of TCM nano/micro delivery systems. (**a**) The impact of microfluidics on the physicochemical properties of NDDSs, and the influences of NDDSs with different structures on experimental results in vivo, reprinted from [39]; (**b**) swelling and degradation properties of MCM reprinted from [105]; (**c**) schematic diagram of nanocomposite hydrogel microspheres that accelerate diabetic wound healing by promoting angiogenesis and reducing inflammatory response, reprinted from [38]; (**d**) graphic presentation of the BCL-LP preparation process by chip, reprinted from [40].

Microfluidic technology affords precise control over the preparation of nano- and micro-scale delivery systems containing TCM active components, enabling high uniformity and tunability in particle-size distribution, drug encapsulation efficiency, and release kinetics [39] (Figure 13a). Traditional methods for TCM nanoparticulate carriers often suffer from broad size distributions and limited batch-to-batch reproducibility. Leveraging laminar flow characteristics and rapid mixing, microfluidic chips allow tight control of particle size and morphology at the microscale, improving monodispersity and encapsulation efficiency [40] (Figure 13d). Microfluidic emulsification strategies for TCM micro-/nano-carriers have attracted increasing attention. Guo and colleagues used microfluidics to construct nanocomposite hydrogel microspheres comprising *Bletilla striata* polysaccharide (BSP) as the matrix and internal ginsenoside PPD nanoliposomes, yielding a “dual-carrier” structure for treating diabetic chronic wounds [38] (Figure 13c). Optimising device parameters produced microspheres with uniform size, tunable mechanics, and good biocompatibility. For oral delivery, Lei and colleagues developed a responsive microcapsule system based on natural polysaccharides from Mesona chinensis; a microfluidic double-emulsion template generated core–shell hydrogel microcapsules [105] (Figure 13b). The system encapsulated *Lactobacillus*, achieving intestinal targeting, with microcapsules controlled within 500–700 µm and a narrow size distribution.

The advantages of microfluidic technology in TCM drug carrier preparation are manifested in multiple aspects. First, through precise control of fluid flow rates, flow rate ratios, and interfacial tension parameters, accurate regulation of drug carrier particle size, morphology, and internal structure can be achieved. Second, by selecting different chip configurations (such as T-junction, flow-focusing, coaxial flow) and solidification mechanisms (photopolymerisation, solvent diffusion, ionic crosslinking), diverse drug carrier forms can be prepared, including nanoemulsions, solid nanoparticles, core–shell microcapsules, and drug-loaded microfibers. This technical flexibility enables researchers to custom-design optimal drug delivery systems based on the physicochemical properties of different TCM components and therapeutic requirements [106,107,108].

#### 2.5.2. Microfluidic Controlled Release and Targeted Delivery Technology

**Figure 14:**
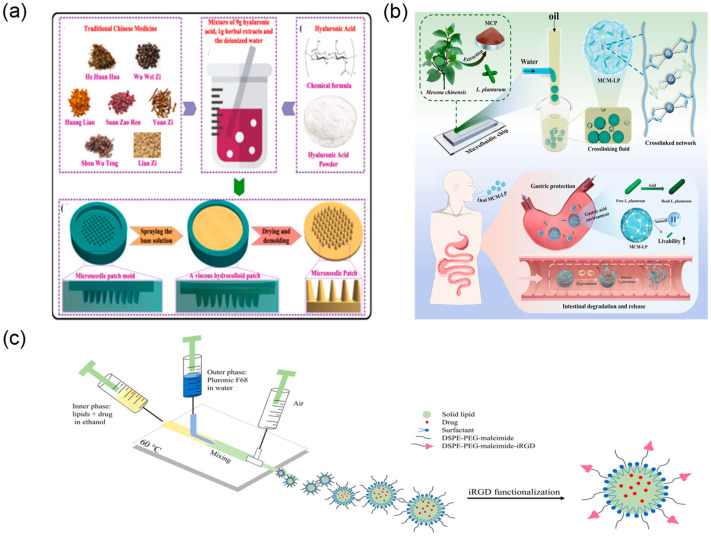
Controlled release and targeted delivery via microfluidic devices. (**a**) Main fabrication process of the TCM DMN patch, reprinted from [109]; (**b**) schematic diagram of MCM preparation, adapted from [105]; (**c**) schematic representation of the setup for producing SLNs using microfluidics and later functionalisation, adapted from [110].

TCM drug delivery systems prepared using microfluidic technology demonstrate exceptional performance in achieving programmable controlled release and targeted delivery of drugs, truly realising the therapeutic strategy of “precise delivery and on-demand release” for TCM active components. In controlled release mechanism design, microfluidic technology endows drug delivery systems with precise control over drug release kinetics through sophisticated structural design and intelligent material selection. Taking the aforementioned pH-responsive microcapsules as an example, they utilise the swelling and degradation characteristics of natural polysaccharides under different pH environments to achieve selective drug release at different gastrointestinal sites [105] (Figure 14b). In-depth drug release kinetic studies indicate that compared to free drugs, the residence time of microcapsule-encapsulated active components at target sites was extended 3–5-fold, with more stable and controllable drug release profiles, effectively avoiding burst release effects. This intelligent controlled release design not only improves therapeutic efficacy but also significantly reduces systemic side effects.

In the field of targeted delivery, microfluidic drug carrier technology provides innovative solutions for achieving precise delivery of TCM active components, enabling specific drug transport to diseased sites while enhancing therapeutic efficacy and significantly reducing systemic toxicity. Nanoparticulate drug delivery systems prepared through microfluidic technology possess highly controllable particle size distribution characteristics. Through surface functionalisation strategies such as ligand–receptor recognition and antibody conjugation, active targeting and selective accumulation at specific tissues or cells can be achieved [110,111] (Figure 14c).

Microfluidic implantable drug delivery devices offer advantages for localised, directional release. Transdermal systems exemplified by microneedle patches deliver TCM extracts directly to diseased skin, avoiding first-pass effects and broad systemic distribution associated with systemic administration [109] (Figure 14a). This localised strategy increases drug concentration at lesion sites and minimises off-target exposure. In addition, research groups are exploring approaches that integrate TCM theory with modern microfluidics. Intelligent microneedle systems informed by meridian theory and acupoint principles aim to regulate organ function via acupoint-specific dosing. This mode combines traditional theory with modern technology, preserving the holistic and syndrome differentiation perspectives of TCM while improving precision and safety through accurate dose control and targeted delivery [112].

As key actuators in microfluidic delivery, micropumps are used in implantable devices and subcutaneous infusion systems. These pumps enable precise control of flow rates and dosing volumes for liquid TCM preparations at µL and even nL scales, supporting continuous infusion and pulsatile administration [113]. The development of microfluidic delivery systems addresses limitations of conventional administration in dose control and temporal regulation [114].

Microfluidic controlled release and targeted delivery technologies enable precision and personalisation in TCM therapy by controlling the timing, location, and dose of release. This concept aligns with the core TCM principle of “syndrome differentiation and individualised treatment,” providing technical support for the use of TCM within modern medical systems. In complex indications—including oncology, diabetic complications, and chronic wound repair—microfluidic-enabled TCM delivery systems showed clinical potential and broad development prospects.

## 3. Future Perspectives

Integrating microfluidics into TCM modernisation holds promise, yet translation to clinical practice requires coordinated progress across technical and regulatory domains. The lack of unified specifications has long impeded the field [115]. The introduction of ISO 22916:2022 provides an important international framework covering dimensional standards, interface protocols, and device classification [116]. From an implementation perspective, deployment of microfluidics in TCM will likely proceed via targeted applications. Quality control is the most accessible entry: on-line and near-line systems can accelerate release testing for heavy metals, pesticide residues, and illegal additives while enabling batch-to-batch fingerprint verification; portable devices for third-party labs and regulators can strengthen market surveillance.

Microfluidic platforms offer opportunities to improve ethical and experimental standards by reducing animal use through early-stage efficacy and toxicity testing in human-relevant in vitro models, aligning with the 3Rs principle (replacement, reduction, and refinement) that underpins modern biomedical research. Nevertheless, several practical factors still constrain their broader implementation in TCM research: the technical complexity of chip fabrication and operation requires specialised equipment and interdisciplinary expertise, limiting accessibility to routine pharmacological and clinical laboratories; strong device dependence and the lack of unified interface standards hinder data interoperability and cross-platform reproducibility; and clinical translation remains challenging because laboratory-scale prototypes must meet stringent quality assurance, sterility, and regulatory requirements for medical devices. Looking forward, deeper integration of artificial intelligence, advanced materials, and nanotechnology with microfluidics is expected to enable end-to-end automation—from sample processing to data analysis—while multi-organ-on-a-chip systems provide physiologically relevant simulations of human systemic responses. These advances could accelerate TCM’s transition from empirical practice toward precision medicine, strengthening the modernisation of traditional pharmaceutical sciences once operation is simplified, costs are reduced, and clinical validation is streamlined.

## Figures and Tables

**Table 1:** Summary of microfluidic architectures and reliability features for TCM quality, safety, and pharmacological evaluation.

Application Area	Representative Microfluidic Architecture	Notes on Reliability and Sustainability	Representative Refs.
Quality analysis—chemical composition and fingerprinting	Microchip electrophoresis; laminar flow extraction; SlipChip serial dilution; lab-on-a-disc	Fixed geometry and steady-state flow facilitate reproducibility; significantly reduces sample/reagent consumption compared to benchtop HPLC.	[19,24,25]
Safety evaluation—pathogens, heavy metals, additives	Optical immunosensor-on-chip; paper-based distance-readout (µPAD); enzyme inhibition microchips	Portable with fewer machines, enabling on-site screening; low reagent and waste levels facilitate large-scale monitoring.	[26,27,28]
Toxicity evaluation	Perfused liver-on-a-chip; droplet single-cell cytotoxicity; neuronal/cardiac tissue chips	Continuous perfusion enhances human relevance; single-cell format reveals rare toxic phenotypes, enhancing predictive value.	[29,30,31]
Pharmacological evaluation	Organoid/organ-on-a-chip; co-culture under flow	Stable nutrition and metabolic clearance reduce cross-repetition variability and improve the accuracy of effect size estimation.	[32,33,34]
High-throughput screening	Droplet microfluidics (merging/encoding) array chip platforms	Conditional barcoding and arraying facilitate reproducibility; unit condition cost is significantly reduced.	[35,36,37]
Drug delivery and release	Microfluidic emulsification; liposomes; core–shell hydrogel microcapsules; nanocomposite microspheres	Chip-based fabrication improves batch-to-batch consistency; reduces solvent and energy consumption.	[38,39,40]

## Data Availability

The data presented in this study are available on request from the corresponding author.
